# Human Adipose-Derived Stem Cells Combined with Nano-Hydrogel Promote Functional Recovery after Spinal Cord Injury in Rats

**DOI:** 10.3390/biology11050781

**Published:** 2022-05-20

**Authors:** Jianping Li, Zhisheng Ji, Yu Wang, Tiantian Li, Jinghua Luo, Jun Li, Xueshuang Shi, Liming Li, Liumin He, Wutian Wu

**Affiliations:** 1 Guangdong-Hong Kong-Macau Institute of CNS Regeneration, College of Life Science and Technology, Jinan University, Guangzhou 510632, China; ljp1112111@sohu.com (J.L.); jizhisheng0521@163.com (Z.J.); 18845724476@163.com (Y.W.); ltt525212022@163.com (T.L.); ljh_jnu@126.com (J.L.); l18894515757@sina.com (J.L.); sxs_jnu@163.com (X.S.); lm13535224597@163.com (L.L.); 2 Department of Human Anatomy, Zhaoqing Medical College, Zhaoqing 526020, China; 3 Department of Human Anatomy, School of Basic Medicine, Zhuhai Campus of Zunyi Medical University, Zhuhai 519041, China; 4 Department of Orthopedics, The First Affiliated Hospital, Jinan University, Guangzhou 510632, China; 5 Spine Surgery, The Third Affiliated Hospital of Sun Yat-Sen University, Sun Yat-Sen University, Guangzhou 510630, China; 6 Re-Stem Biotechnology Co., Ltd., Suzhou 215129, China

**Keywords:** adipose mesenchymal stem cells, nano-hydrogel, RADA16-RGD, RADA16-I, spinal cord injury, transplantation

## Abstract

**Simple Summary:**

Nerve regeneration and functional recovery after spinal cord injury (SCI) are worldwide problems. Scientists have achieved encouraging results in the repair of spinal cord injuries using natural or synthetic materials. In this paper, we report that nano-hydrogel combined with human adipose-derived stem cells regulate the inflammatory microenvironment, protect neurons and axons, and promote motor function recovery. In addition, three proteins related to neuronal and axonal growth were screened by Liquid chromatography-mass spectrometry. These results provide evidence for clinical treatment of spinal cord injury.

**Abstract:**

The treatment of spinal cord injury aims to reconstruct the fiber connection and restore the interrupted neural pathways. Adipose mesenchymal stem cells (ADSCs) can promote the recovery of motor functions in spinal cord injury. However, poor survival of ADSCs and leakage outside of the injury site after local transplantation reduce the number of cells, which seriously attenuates the cumulative effect. We performed heterotopic transplantation on rats with severe spinal cord injury using human ADSCs loaded within self-assembly hydrogel RADA16-RGD (R: arginine; A: alanine; D: aspartic acid; G: glycine). Our results indicate that the combined transplantation of human ADSCs with RADA16-RGD improved the survival of ADSCs at the injured site. The inflammatory reaction was inhibited, with improved survival of the neurons and increased residual area of nerve fibers and myelin protein. The functional behaviors were promoted, as determined by the Basso, Beattie, and Bresnahan (BBB) locomotor rating scale score and electrophysiological measurements. ADSCs can promote the repair of spinal cord injury. This study provides new ideas for the treatment of spinal cord injury.

## 1. Introduction

Spinal cord injury (SCI) usually leads to paralysis because of motor and sensory dysfunction below the injury level [1], leading to muscle atrophy, thrombosis, joint deformation, pulmonary embolism, repeated urinary tract infection, dysphagia, and pain, among others [2,3,4,5]. These complications seriously affect the physical and mental health of patients and lower their quality of life. Falls, followed by traffic accidents and impact from falling objects have been reported as the primary causes of SCI [6,7]. SCI can be devastating to the affected individual as well as have an adverse effect on the family owing to the huge societal cost involved [8]. Currently, there is no effective treatment for SCI.

Glial cells, leukocytes, pro-inflammatory cytokines (tumor necrosis factor α, TNF-α; interleukin-1β, IL-1β; and interleukin-6, IL-6), and some active substances (such as reactive oxygen species, kinin, histamine, and nitric oxide) constitute the immune microenvironment after SCI [9,10,11,12]. In addition, the resultant injury destroys the spinal cord structure, leading to ischemia and hypoxia as well as the formation of a cavity surrounded by a glia fiber scar. Moreover, the cells in the scar secrete a variety of chemicals that inhibit the growth of axons. Therefore, it is important to improve the post-sci regeneration microenvironment, fill the spinal cavity, and promote axon regeneration. Biological scaffolds play an important role in the repair of SCI. The structural properties of the scaffold material can fill the cavity, regulate the microenvironment of the damaged area, and promote the regeneration of axons as well as the recovery of animal behavioral function [13]. Biological materials for scaffolds include natural and synthetic materials that can be processed into a guide channel scaffold with a porous structure, acellular scaffold structure, or hydrogel [14]. The spinal cord is a soft, watery, hydrogel-like biological structure. Accordingly, hydrogel exhibits unique advantages in the treatment of SCI. RADA16-I is a peptide formed by the four repeats of R, A, D, and A amino acid residues (R: arginine; A: alanine; D: aspartic acid), which can self-assemble into a three-dimensional gel structure composed of nanofibers at a pH of 7.4 [15]. RADA16-I can be used as a drug carrier and for tissue engineering scaffolds, with wide applications in biomedical engineering. Numerous studies have reported that the C-terminus of the RADA16-I self-assembled oligopeptide can bind RGD (R: arginine; D: aspartic acid; G: glycine), IKVAV (I: isoleucine; K: lysine; V: valine; A: alanine), and PDSGR (P: proline; D: aspartic acid; S: serine; G: glycine; A: arginine) [16]. The bioactive peptide fragment endows the structure with bioactivity similar to that of the original peptide fragment. These studies provide a theoretical basis for research on self-assembled nanofiber materials with specific functions and their subsequent application in tissue regeneration. The present investigation revealed that RADA16-RGD promotes the proliferation of neural stem cells, differentiation into neurons and astrocytes, and elongation of neurites [17].

Stem cell transplantation is one of the most important therapeutic strategies for SCI. The efficacy of stem cells has been verified by several animal model studies and several recent relevant clinical studies [18,19,20]. The transplantation of cells that secrete anti-inflammatory cytokines has been documented to promote axonal regeneration after SCIs, such as Schwann cells, bone marrow mesenchymal stem cells (MSCs), umbilical cord MSCs, and adipose MSCs (ADSCs) [21,22,23]. Among the cells available for transplantation, ADSCs have become a research hotspot for tissue repair owing to their wide distribution, easy access, low immune rejection, and high safety [24,25]. Past studies have demonstrated that the mechanism of MSCs in the treatment of SCI is to inhibit inflammation, improve the injury microenvironment, protect the functions of the remaining nerve tissues, stimulate the plasticity of the host nervous system, and promote the reconstruction of the compensatory circuit of undamaged neurons in order to achieve the effect of functional repair [20,26,27]. However, the mechanism by which the transplanted adipose stem cells interact with the endogenous cells to promote spinal cord nerve regeneration remains unclear.

In this study, human adipose tissue-derived stem cells loaded with RADA16-RGD hydrogel were transplanted into rat models of SCI to study the subsequent improvement in the survival of the transplanted cells. We also investigated the mechanism by which RADA16-RGD combined with ADSCs to promote SCI repair.

## 2. Materials and Methods

### 2.1. Establishment of an SCI Model

All animal experiments were approved by the Laboratory Animal Ethics Committee at Jinan University, China (approval no. 20180228026) on 18 February 2018. Female Sprague Dawley (SD) rats (weight: 220–250 g) were provided by Guangdong Medical Experimental Animal Center (Guangzhou, China; license no. SYXK (Yue) 2017-0174). Pentobarbital sodium (32 mg/kg; Sinopharm Chemical Reagent Co., Ltd., Beijing, China) was injected intraperitoneally into the rats to induce anesthesia. The T10 segment was fixed with a spinal cord fixator. The lamina of T10 was removed with a rongeur to expose the spinal cord. The rats with exposed spinal cord were then fixed on the impact platform of the SCI system impactor Louisville Injury System Apparatus (LISA, Louisville, KY, USA). The impact parameters were set as follows: the diameter of the impact head was kept at 2.2 mm, the impact depth at 1.4 mm, and the residence time at 0.6 s. The spinal cord was observed again after the impact. Rats were excluded if the dura mater ruptured and the spinal cord protruded beyond the dura along the rupture. After the surgery, the rats were provided with special nursing and clean drinking water. The rats were then subcutaneously injected with gentamicin (240 IU/mL, Guangdong Bangmin Pharmaceutical, Jiangmen, China), 2 mL/time, and 2 times/day for 7 consecutive days. Bladder compressions were performed every 8 h for urination until the rats could spontaneously micturate. The sample size of rats in this experiment are described in Supplementary Table S1.

### 2.2. Preparation of ADSCs+RADA16-RGD Self-Assembly Hydrogel

RADA16-RGD (2%) solution was prepared with double distilled water and then filtered after complete dissolution. The filtrate was adjusted to pH 7.4 with Tris-HCl buffer and allowed to settle for 5 min. Finally, the ADSCs (Saliai Stem Cell Science and Technology Co., Ltd., Guangzhou, China) and RADA16-RGD were fully mixed and configured into ADSCs+RADA16-RGD (1 × 10^5^ cell/µL).

### 2.3. Implantation of ADSCs+RADA16-RGD

The Basso, Beattie, and Bresnahan (BBB) score was recorded on the second week of SCI. The rats with the difference in the left and right hind limb scores of ≥3 or an average score of both the hind limbs > 3 were excluded. The rats were randomly divided into PBS, ADSCs, RADA16-RGD, and ADSCs+RADA16-RGD groups.

After exposing the spinal cord, transplantation was performed at the center of the injury site on the 14th day of SCI. Then, 10 µL of ADSCs+RADA16-RGD was injected using the microinjector (Cat. No. 1701; Hamilton, Bonaduz, Switzerland), with a 40 µm diameter needle (glass electrode; Cat. No. 4878; World Precision Instruments, Sarasota, FL, USA). The microinjector was inserted to 1.5 mm depth and then drawn back to 1.2 mm. After a slow injection of 5 µL of ADSCs+RADA16-RGD, the needle was retained for 5 min at the site and then drawn back by 0.4 mm. After a slow injection of the remaining solution, the needle was retained for 5 min and then slowly withdrawn. The ADSC groups were injected with ADSCs (10 µL, 1 × 10^5^ cell/µL). The rats in the RADA16-RGD and PBS groups were injected with 10 µL of RADA16-RGD and PBS, respectively. The rats in all groups were subcutaneously injected with cyclosporine A (15 mg/kg; Novartis, Basel, Switzerland) once a day for 8 weeks and then with gentamicin twice a day for 7 days after the surgery.

### 2.4. Flow Cytometry

The P5-generation ADSC suspension was centrifuged at 1000 rpm for 5 min, and the supernatant was discarded. After adding the dye buffer, the mixture was centrifuged at 4 °C and 1000 rpm for 5 min, and the supernatant was discarded. The same volume of dyeing buffer from the previous step was added for resuspension, and then the suspended solution was incubated in a refrigerator at 4 °C for 20 min. After adding the dye buffer, the aforementioned procedure was repeated once. The cells were then resuspended in the loading buffer. The suspension was filtered through a sieve and transferred to a flow tube for flow cytometric analyses (Beckman, DxFLEX, Indianapolis, IN, USA).

### 2.5. Induction and Differentiation Culture of ADSCs

#### 2.5.1. Osteogenic/Adipogenic Differentiation

The P5-generation ADSCs were implanted into 24-well plates (2 × 10^4^ cells/well) for osteogenic (Cat. No. G03012; Guangzhou Celera Stem Cell Technology Co., Ltd., Guangzhou, China)/adipogenic (Cat. No. G03013; Guangzhou Celera Stem Cell Technology Co., Ltd., Guangzhou, China) differentiation for 3–4 weeks according to the manufacturer’s protocol. Alizarin red staining and Oil red O staining were performed for osteogenic and adipogenic differentiation, respectively. The cells were dyed with Alizarin red solution (1 mL/well) for 20 min. The Oil Red O solution (A:B = 5:2) was then mixed and filtered, followed by dyeing for 30 min.

#### 2.5.2. Chondrogenic Differentiation

The suspension containing 3 × 10^5^ P5-generation ADSCs was centrifuged at 300× *g* for 5 min. The centrifugation tube cover was loosened and chondrogenesis (Cat. No. Abs9406; Absin, Shanghai, China) was induced for 21 days. After differentiation, the cells were stained with safranine O for 1–2 h.

#### 2.5.3. BBB Score and Motor-Evoked Potential Monitoring

Until sampling, the BBB score of the animals’ hind limb motor function was recorded in an open field every week after the surgery, for up to 10 weeks [28]. Both the left and right hind limbs were scored by two people, and the entire process was videotaped. The scores of the left and right hind limbs were averaged for further calculation.

### 2.6. Detection of Exercise-Induced Electrophysiology

The rats were injected with pentobarbital sodium for abdominal anesthesia 8 weeks after the transplantation treatment. The head of the rats was fixed on a stereotactic apparatus (Cat. No. 69101; RWD, Shenzhen, China). The anterior fontanel was taken as the origin, with 2 mm downward and 1.5 mm from both sides. The cranial opening measured 2 × 2 mm^2^. The stimulating electrode of the BL-420 biological function experimental system (Tme, Chengdu, China) was attached to the motor cortex of rats, and the receiving electrode was placed on the contralateral gastrocnemius muscle with a single stimulus. The stimulation voltage was 10 V, the interval time was 0.1 s, and the detection time was 2 min. The latency and amplitude of the rats in each group were recorded.

### 2.7. Cell Culture

The spinal cord tissue of newborn (day 1) Sprague Dawley rats was digested with accutase (Cat no. A6964; Sigma; St. Louis, MO, USA) and filtered through a 200 µm mesh filter. After centrifugation at 800× *g*, the cells were suspended in an ADSCs complete medium (Cat no. CM-H112; Procell Life Science & Technology Co., Ltd.; Guangzhou, China) and cultured in a 14 mm diameter cell culture slide. The cell cultures were divided into 3 groups: the control group (spinal nerve cells), the RADA16-RGD group (spinal nerve cells + RADA16-RGD), and the ADSCs+RADA16-RGD group (spinal nerve cells+ADSCs+RADA16-RGD). ADSCs were seeded at a density of 4 × 10^4^/well, and the density of the spinal nerve cells was 5 × 10^4^/well. The culture medium was changed the following day, and thereafter every 3 days. The cells were cultured for 7 days. From the resultant mixed culture, the spinal cord cells were stained with an anti-tubulin beta-3 chain (anti-TUJ-1) antibody. The cell culture slide was divided into 3 equal portions, and the number of TUJ-1^+^ cells in the middle-third portion of the slides was counted. To assess the neurite length, 5 locations in the cell counting area of the slide were randomly selected. Two neurons were selected from each position, and the straight-line distance from the neurite origin at the cell body to its end was measured and analyzed using the software provided by the Zeiss microscope (Imager Z2 Zeiss).

### 2.8. Immunofluorescence Staining

The rats were anesthetized through intraperitoneal injection with 1 mL of 10% chloral hydrate and perfused with 4% paraformaldehyde. A total length of 1.8 cm of the spinal cord was taken, with 0.9 cm above and below the injury center. The slice thickness was 16 µm. A slice was sampled every 0.5 mm and 128 µm in the coronal section and sagittal section, respectively. The slices were incubated with the primary antibodies at room temperature for 2 h and then placed at 4 °C overnight, followed by incubation with the secondary antibodies at room temperature for 2 h.

### 2.9. Immunofluorescence Analysis

Surviving ADSC count: Human nuclear (HUNA)-positive cells were counted by using ImageJ software at 1, 2, 3, and 4 weeks after the transplantation using the Abercrombie formula. The ratio of the number of HUNA-positive cells to the total number of transplanted cells indicates the survival rate of the transplanted cells.

Cell count: Neuronal nuclei (NeuN^+^)-positive cells, choline acetyltransferase-positive cells (ChAT), and ionized calcium-binding adapter molecule 1-positive cells (Iba1^+^) were counted by a double-blind method 8 weeks after the transplantation.

Iba1^+^ cell count: Iba1^+^ cells and activated Iba1^+^ cells were counted in a 5 × 5 mm^2^ area from either side above the central canal of the spinal cord at the 4 mm head side of the injury center. The ratio of activated Iba1^+^ cells divided by the total number of Iba1^+^ cells indicates the activation rate of Iba1^+^ cells.

Myelin basic protein (MBP) immunofluorescence density: Images of the entire transverse sections stained for MBP 8 weeks after stem cell transplantation were captured with consistent exposure time under the same conditions with a 20X objective using the Zen tiling (imager Z2 Zeiss). Immunoreactive density was measured using the ImageJ software.

Nerve fiber count: The spinal cord was harvested and sagittally transected 8 weeks after transplantation. A line perpendicular to the long axis of the spinal cord was drawn at the center of the injury, and neurofilament 200 nerve fibers (NF200^+^) passing through the line (fiber length > 40 μm) were counted by a double-blind method. A line perpendicular to the long axis of the spinal cord at the center side was drawn for counting the 5-hydroxy tryptamine (5-HT) nerve fibers. The counting method was the same as that used for the NF200^+^ nerve fibers.

### 2.10. ELISA

The spinal cord of rats was harvested 2 weeks after the transplantation, the fragmented tissue was ground, and the supernatant was collected after centrifugation. The levels of TNF-α, IL-1β, and IL-6 were measured using an ELISA kit as per the manufacturer’s protocol.

### 2.11. Western Blotting

Proteins (40 µg/well) were electrophoresed on sodium dodecyl sulfate–polyacrylamide gel electrophoresis (SDS-PAGE, Cat no. SK6010-250T; Coolaber) and transferred onto nitrocellulose membranes (Cat no. 10600023; Cytiva). The membranes were blocked with 5% skim milk at room temperature for 1.5 h. The protein levels were analyzed by probing with specific primary antibodies against pleiotrophin (recombinant rabbit antibody PTN), fibroblast growth factor-1 (recombinant rabbit antibody FGF1), and nestin (recombinant rabbit antibody), which were incubated with the blots at 4 °C overnight, followed by incubation with goat anti-rabbit horseradish peroxidase (HRP)-conjugated antibody for 1 h at room temperature. The protein bands were detected by enhanced chemiluminescence, and ImageJ software was used for optical density analysis.

### 2.12. Liquid Chromatography (LC)–Mass Spectrometry (MS)

To further explore the potential molecular mechanism of ADSCs+RADA16-RGD action in SCI repair, we performed LC-MS analysis on spinal cord tissues 1.8 cm around the injured site in both the ADSCs+RADA16-RGD and PBS groups after 2 weeks of transplantation. LC-MS (Ultimate 5600, AB SCIEX) analysis was performed as previously described [29]. Proteins in the spinal cord tissue samples were first parsed on 4–12% NuPAGE gel and then visualized with a colloid blue staining kit; glutathione S-transferase (GST)–spastin pull-down assays were then performed by LC-MS. A high-performance LC Ultimate 5600 system was employed to analyze the peptides obtained from trypsin digestion by nanoflow reversed-phase LC-tandem MS coupled with linear ion-trap data acquisition mode.

Heat map generation and clustering: The enrichment degree of the differential proteins identified by LC-MS in Gene Ontology (GO) terms was analyzed and used to prepare heat maps based on their log2 (transected/sham) fold-change values. The “Correlation (Centered)” similarity measure in cluster 3.0 was employed for hierarchical clustering. The Java TreeView was used to generate the heat map from the output.cdt file. The differentially expressed proteins were evaluated by the Kyoto Encyclopedia of Genes and Genomes (KEGG) and principal component analysis [30].

### 2.13. Statistical Analysis

GraphPad Prism 6 software was used for statistical analysis. The results are represented as mean ± standard error of the mean (SEM). The variance homogeneity was measured between the two groups for the measurement data. If the variance was homogeneous, the *t*-test was performed; otherwise, the *t*-test was performed after the correction. The counting data were analyzed using the *χ*^2^ test.

## 3. Results

### 3.1. Identification of ADSCs and the Survival of Transplanted Cells

Flow cytometry analysis of the fifth-generation ADSCs exhibited that the expression rates of MSC-positive markers CD73, CD90, and CD105 exceeded 95%, while those of cell-negative markers CD11b, CD19, CD34, CD45, and HLA-DR were <2%, indicating a high cell purity (Supplementary Figure S1A). The cells exhibited long fusiform cell bodies with elongated processes. Adipose stem cells have multidirectional differentiation potential. Hence, we induced differentiation and demonstrated that the adipose stem cells differentiated into adipocytes, bone cells, and chondrocytes (Supplementary Figure S1B–E).

### 3.2. ADSCs+RADA16-RGD Increased the Neuron Numbers and Axon Lengths In Vitro

**Figure 1:**
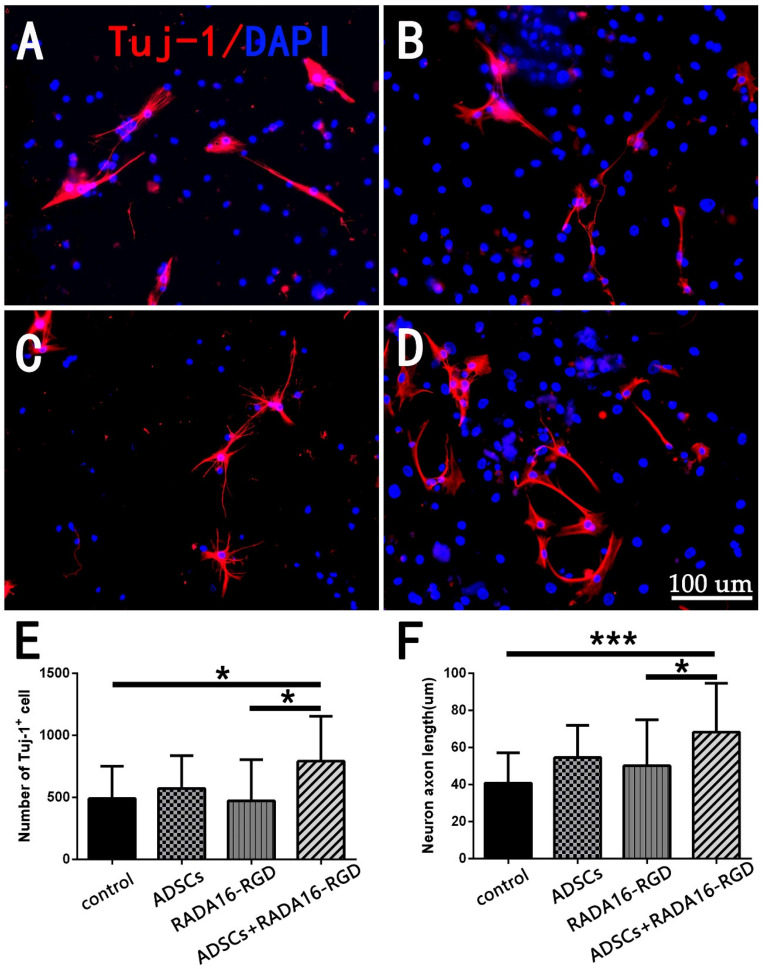
Detection of the numbers and neurite lengths of cultured TUJ-1^+^ spinal cord neurons. TUJ-1 antibody staining after culturing neurons for 7 days. (**A**) Control group. (**B**) RADA16-RGD group. (**C**) ADSCs group. (**D**) ADSCs+RADA16-RGD group. (**E**) The number of TUJ-1^+^ neurons in the ADSCs+RADA16-RGD group was significantly greater than that in the control or RADA16-RGD groups (ADSCs+RADA16-RGD vs. control or RADA16-RGD: *p* < 0.05); however, there was no significant difference between the ADSCs and ADSCs+RADA16-RGD groups (*p* > 0.05), and the control, ADSCs, and the RADA16-RGD groups showed no significance (*p* > 0.05). (**F**) The axonal lengths in the ADSCs+RADA16-RGD group were significantly greater than in the control or RADA16-RGD groups (ADSCs+RADA16-RGD vs. control or RADA16-RGD: *p* < 0.05); however, there was no significant difference between the ADSCs and ADSCs+RADA16-RGD groups (*p* > 0.05), and the control, ADSCs, and the RADA16-RGD groups showed no significance (*p* > 0.05). Data are presented as the mean ± SEM (*n* = 4 per group). Scale bar = 100 µm. * *p* < 0.05, *** *p* < 0.001. TUJ-1: βIII-tubulin; ADSCs: adipose stem cells; DAPI: 4′ 6-diamidino-2-phenylindole. TUJ-1 (red): Alexa Fluor 647.

Neuronal survival and axonal regeneration are essential for SCI repair. To investigate whether ADSCs+RADA16-RGD could promote neuron survival and axonal growth, we assessed the neuronal numbers and axonal lengths after the spinal cord neurons had been cultured in vitro for 7 days with the TUJ-1 antibody. The number of TUJ-1^+^ neurons and the axonal lengths in the ADSCs+RADA16-RGD group were significantly greater than in the control or RADA16-RGD groups (Figure 1). This result denotes that ADSCs+RADA16-RGD could improve neuron survival and promote axonal growth.

### 3.3. Survival of Transplanted Cells

**Figure 2:**
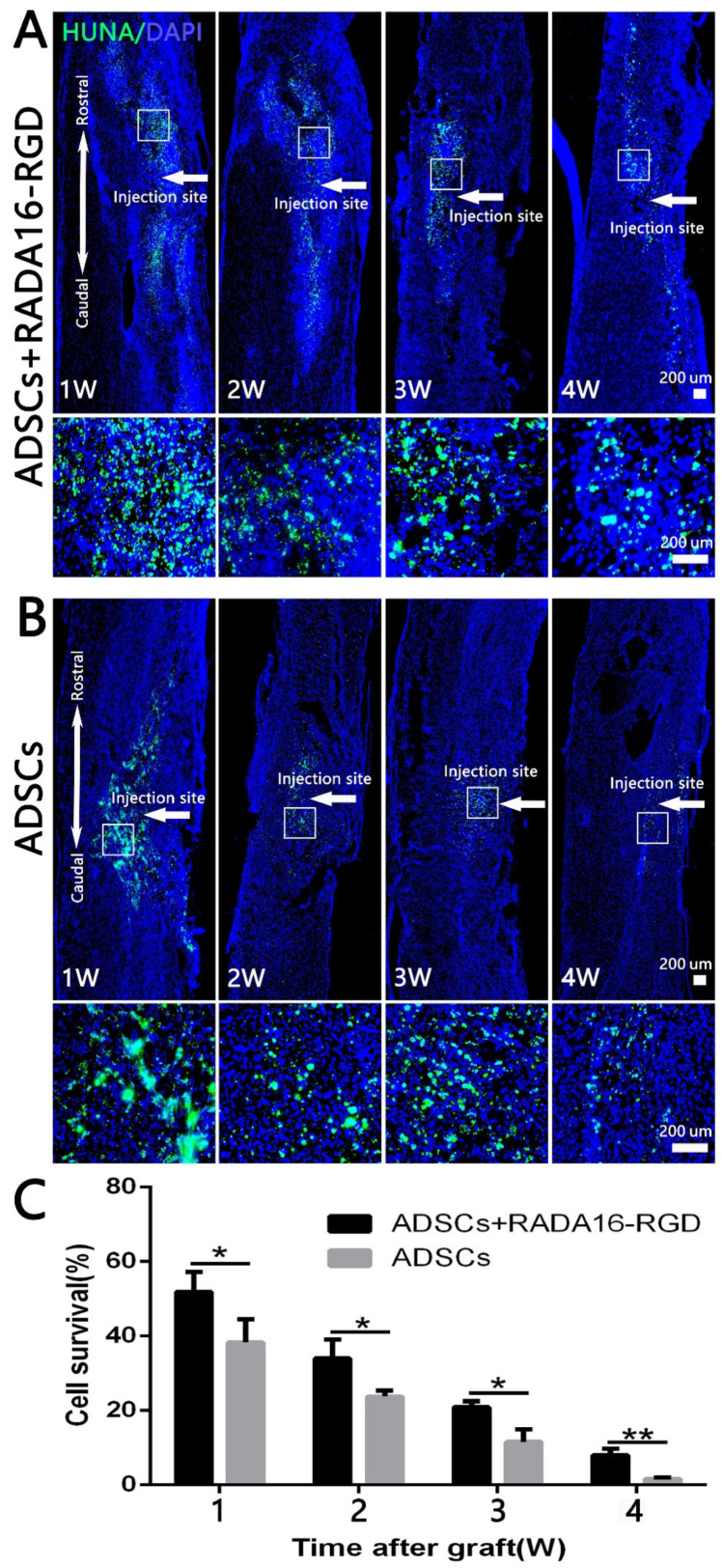
ADSC survival at different time points after transplantation. HUNA antibody staining was performed in the spinal cord at 1–4 weeks after adipose stem cell transplantation, and the HUNA^+^ cells were distributed at the injury site around the injection point (arrow). (**A**) ADSCs+RADA16-RGD group. (**B**) ADSCs group. In both the groups, a large number of HUNA^+^ cells were observed in the first 1–3 weeks after the ADSCs transplantation, and there were fewer HUNA^+^ cells in the 4th week. The panels below (**A**,**B**) show high magnification of the boxed regions in the above panels. (**C**) The amount of surviving adipose stem cells decreased over the lapse of transplantation time in two groups. The ADSC survival rate in the ADSCs+RADA16-RGD group at 1, 2, 3, and 4 weeks was significantly higher than that in the ADSCs group at 1, 2, 3, and 4 weeks. * *p* < 0.05, ** *p* < 0.01. Data are presented as mean ± SEM (*n* = 3 per time point). Scale bar = 200 µm. HUNA: human nuclear antibody; ADSCs: adipose stem cells; DAPI: 4′ 6-diamidino-2-phenylindole. HUNA (green): Alexa Fluor 488.

To assess the survival of the transplanted cells, we prepared the sections at 1–4 weeks and then performed anti-HUNA immunostaining. The spinal cord samples were collected at different time points after the transplantation of ADSCs-RADA16-RGD and ADSCs. Anti-HUNA immunostaining revealed that the ADSCs in the ADSCs-RADA16-RGD group were distributed around the injury site (Figure 2A), while those in the ADSCs group were partially migrated to the spinal cord surface (Figure 2B (1W,2W)). The results of the quantitative analysis suggested that the survival rate of ADSCs in the ADSCs-RADA16-RGD group was significantly greater than that in the ADSCs group (Figure 2C).

### 3.4. Transplantation of ADSCs+RADA16-RGD Improved the Functional Recovery of SCI

**Figure 3:**
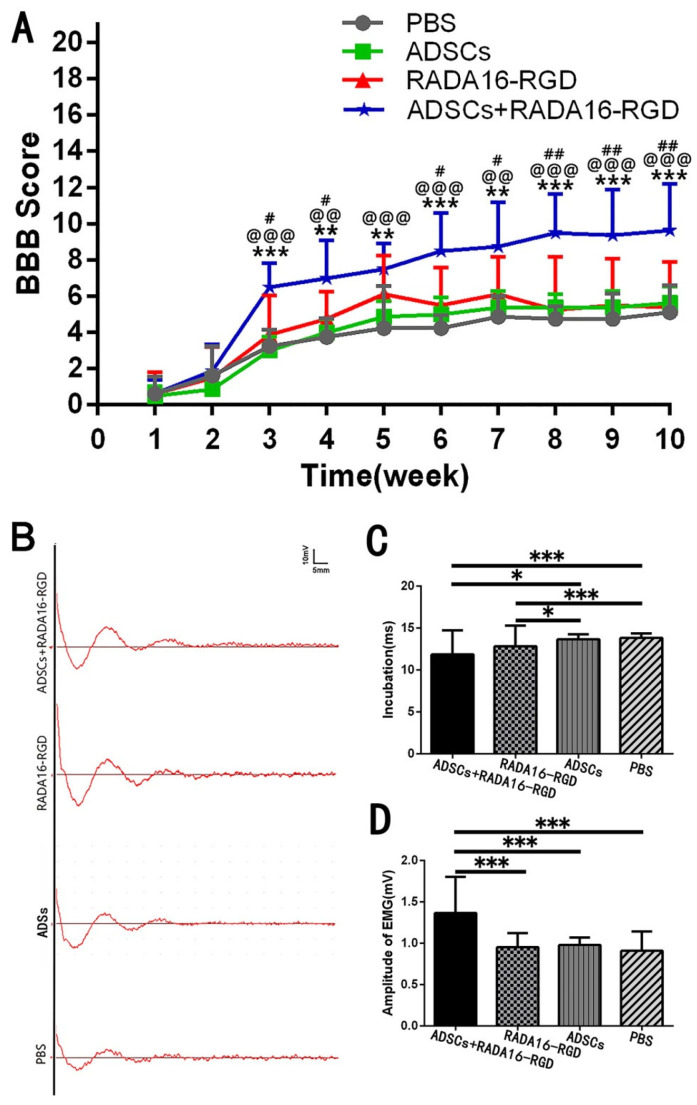
ADSCs+RADA16-RGD transplantation improves motor function in rats. (**A**) BBB scores for 10 consecutive weeks after spinal cord injury. At weeks 1 and 2, when compared with those for the RADA16-RGD group and PBS group, the BBB scores for the ADSCs+RADA16-RGD group showed no significant difference. From 3 to 10 weeks, the ADSCs+RADA16-RGD group’s BBB score increased significantly when compared with those of the PBS, ADSCs, and RADA16-RGD groups (ADSCs+RADA16-RGD vs. PBS: *p* < 0.01 after 4, 5, and 7 weeks, *p* < 0.001 after 3, 6, 8, 9, and 10 weeks; ADSCs+RADA16-RGD vs. ADSCs: *p* < 0.01 after 4 and 7 weeks, *p* < 0.001 after 3, 5, 6, 8, 9, and 10 weeks; ADSCs+RADA16-RGD vs. RADA16-RGD: *p* < 0.05 after 3, 4, 6, and 7 weeks, *p* < 0.01 after 8, 9, and 10 weeks). # ADSCs+RADA16-RGD vs. RADA16-RGD, ^@^ ADSCs+RADA16-RGD vs. ADSCs, * ADSCs+RADA16-RGD vs. PBS. (**B**) MEP records at 8 weeks after transplantation. (**C**) Myoelectric detection indicated that the latency was shorter in the ADSCs+RADA16-RGD and RADA16-RGD groups than in the PBS and ADSCS groups (ADSCs+RADA16-RGD vs. RADA16-RGD: *p* < 0.001, ADSCs+RADA16-RGD vs. ADSCs: *p* < 0.05, RADA16-RGD vs. RADA16-RGD: *p* < 0.001, RADA16-RGD vs. ADSCs: *p* < 0.05). (**D**) The amplitude was significantly higher in the ADSCs+RADA16-RGD group than in the PBS, ADSCs, and RADA16-RGD groups (ADSCs+RADA16-RGD vs. PBS: *p* < 0.001, ADSCs+RADA16-RGD vs. ADSCs: *p* < 0.001, ADSCs+RADA16-RGD vs. RADA16-RGD: *p* < 0.001). Data are presented as mean ± SEM (BBB: *n* = 8 per groups, MEP: *n* = 4 per groups). # *p* < 0.05, ## *p* < 0.01, ^@@^ *p* < 0.01, ^@@@^ *p* < 0.001, ** *p* < 0.01, *** *p* < 0.001. Data are presented as mean ± SEM (BBB score *n* = 8 per group, MEP *n* = 4 per group). ADSCs: adipose stem cells; BBB: Basso, Beattie, and Bresnahan scale; MEP: motor-evoked potential; EMG: electromyogram.

The BBB score was used to evaluate the recovery of hind limb motor function at different time points after SCI. Joint movement, coordination of limb movement, and trunk stability were evaluated. The BBB score of normal rats was 21. The scores of the three groups increased with time after the transplantation, and the motor function was most obviously recovered 3 weeks after the injury. The score was significantly higher in the ADSCs+RADA16-RGD group than in the PBS, ADSCs, or ADSCs+RADA16-RGD groups. No significant difference was found in the score between the PBS, ADSCs, and RADA16-RGD groups (Figure 3A).

To verify the effect of ADSCs+RADA16-RGD on the functional recovery in the rats, electromyography was performed 8 weeks after transplantation for SCI. One side of the motor cortex was stimulated, and the electrical signal in the gastrocnemius muscle on the opposite side was assessed. The functional recovery of rats with SCI was evaluated based on the electromyography waveform and amplitude. Myoelectric detection was accomplished 8 weeks after the transplantation in rats with SCI. The motor cortex was stimulated to receive signals from the contralateral gastrocnemius muscle. Myoelectric detection indicated that the latency was significantly decreased. The amplitude was significantly higher (Figure 3B–D).

### 3.5. Transplantation of ADSCs+RADA16-RGD Improved the Survival of Neurons after SCI

**Figure 4:**
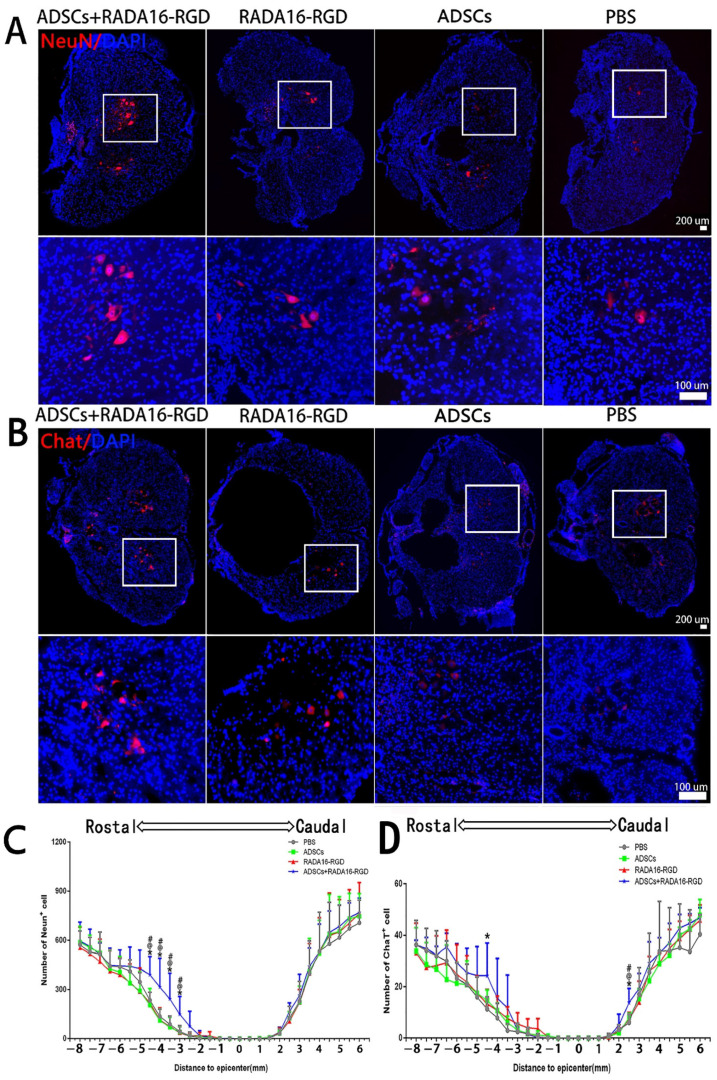
ADSCs+RADA16-RGD transplantation contributes to neuronal survival in rats with spinal cord injury. (**A**) NeuN antibody staining (red) at −4.5 mm cephalic to the injury at 8 weeks after PBS, ADSCs, RADA16-RGD, and ADSCs+RADA16-RGD transplantation. (**B**) ChAT antibody staining (red) at −4.5 mm cephalic to the injury at 8 weeks after ADSCs+RADA16-RGD transplantation. The panels below (**A**,**B**) show high magnification of the boxed regions in the abovementioned panels. Scale bar = 200 µm, high magnification regions scale bar = 200 µm. (**C**) Quantitative analysis of NeuN^+^ cells transected at different distances from the injury center. The number of NeuN^+^ neurons on the cephalic side of the injury center was significantly higher in the ADSCs+RADA16-RGD group than in the PBS, ADSCs, and RADA16-RGD groups (ADSCs+RADA16-RGD vs. PBS: *p* < 0.05, ADSCs+RADA16-RGD vs. ADSCs: *p* < 0.05, ADSCs+RADA16-RGD vs. RADA16-RGD: *p* < 0.05, at −3 mm, −3.5 mm, −4 mm, and −4.5 mm). (**D**) Quantitative analysis of ChAT^+^ cells transected at different distances from the injury center. The number of ChAT^+^ neurons was greater in the ADSCs+RADA16-RGD group than in the PBS group at −4.5 mm on the cephalic side of the injury center (*p* < 0.05). Similarly, the number of ChAT^+^ neurons was greater in the ADSCs+RADA16-RGD group than in the PBS, ADSCs, and RADA16-RGD groups at 3 mm (ADSCs+RADA16-RGD vs. PBS: *p* < 0.05, ADSCs+RADA16-RGD vs. ADSCs: *p* < 0.05, ADSCs+RADA16-RGD vs. RADA16-RGD: *p* < 0.05) on the caudal side of the injury center. *: ADSCs+RADA16-RGD vs. PBS, ^@^: ADSCs+RADA16-RGD vs. ADSCs, # ADSCs+RADA16-RGD vs. RADA16-RGD. * *p* < 0.05, ^@^ *p* < 0.05, # *p* < 0.05. Data are presented as the mean ± SEM (NeuN *n* = 6 per group, ChAT *n* = 6 per group). ADSCs: adipose stem cells; DAPI: 4′6-diamidino-2-phenylindole; NeuN: neuronal nuclei; ChAT: choline acetyltransferase. NeuN (red): Alexa Fluor 647, ChAT (red): Alexa Fluor 647.

The present results suggest that the addition of ADSCs+RADA16-RGD could increase the number of surviving neurons after SCI. To identify whether ADSCs+RADA16-RGD exerted the same effect on rats with SCI, the total number of NeuN^+^ and ChAT^+^ neurons was counted in the spinal cord of rats 8 weeks after the transplantation. Few NeuN^+^ neurons survived within 3 mm of the injury center. The number of NeuN^+^ neurons on the cephalic side of the injury center was significantly higher at −3 mm, −3.5 mm, −4 mm, and −4.5 mm (Figure 4A,C). The number of ChAT^+^ neurons was significantly higher at −4.5 mm on the cephalic side and 2.5 mm on the caudal side of the injury center (Figure 4B,D).

### 3.6. Transplantation of ADSCs+RADA16-RGD Increased the Myelin Sheath Area

**Figure 5:**
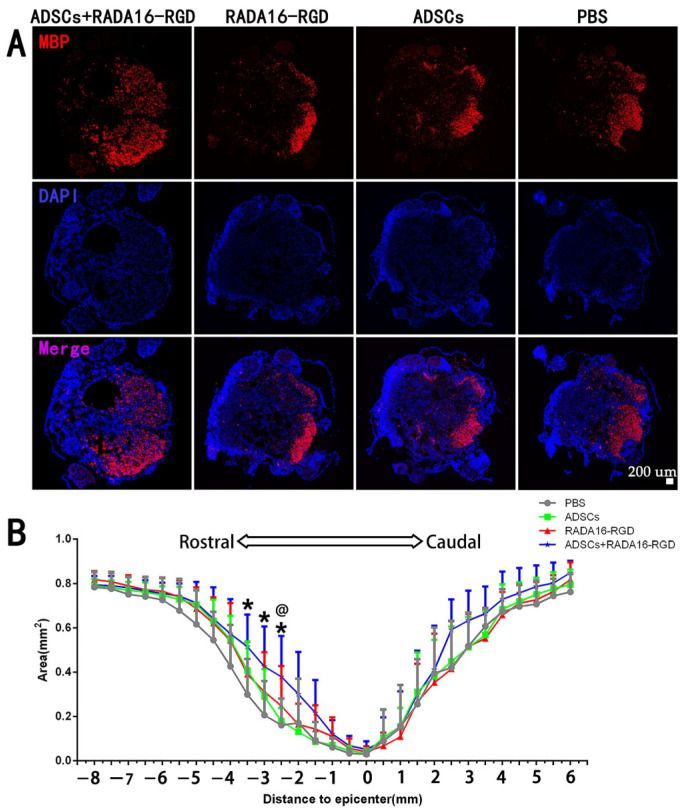
ADSCs+RADA16-RGD transplantation inhibits axonal demyelination in rats with spinal cord injury. (**A**) At 8 weeks after ADSCs+RADA16-RGD transplantation, the area of MBP^+^ region at −2.5 mm rostral to the injury in spinal cord cross-sectional MBP antibody staining (red) was greater in the ADSCs+RADA16-RGD group than in the RADA16-RGD and PBS groups. (**B**) Quantitative analysis of MBP at different distances from the injury center. There were significantly more MBP^+^ areas in the ADSCs+RADA16-RGD group than in the PBS group at −2.5 mm, −3 mm and −3.5 mm on the cephalic side of the injury center (*p* < 0.05); at −2.5 mm, there were significantly more MBP^+^ areas in the ADSCs+RADA16-RGD group than in the ADSCs group (ADSCs+RADA16-RGD vs. PBS or ADSCs: *p* < 0.05) ^@^ ADSCs+RADA16-RGD vs. ADSCs, * ADSCs+RADA16-RGD vs. PBS. ^@^ *p* < 0.05, * *p* < 0.05. Data are presented as mean ± SEM (*n* = 6 per group). Scale bar = 200 µm. ADSCs: adipose stem cells; MBP: myelin basic protein; DAPI: 4′6-diamidino-2-phenylindole. MBP (red): Alexa Fluor 647.

MBP is expressed exclusively in oligodendrocytes, and it plays a critical role in maintaining the structural integrity and functional stability of the medullary sheath. This protein is essential for promoting the remyelination of axons in rats after SCI. The results of myelin marker MBP staining signified that the areas of MBP^+^ at −2.5 mm, −3 mm, and −3.5 mm on the cephalic side of the injury center were significantly larger. The MBP^+^ area in the injury center was larger in the ADSCs+RADA16-RGD group than in the PBS, ADSCs, and RADA16-RGD groups, albeit without any significant difference. Furthermore, no significant difference was recorded in the caudal side of the injury among the three groups (Figure 5).

### 3.7. Transplantation of ADSCs+RADA16-RGD Increased the Local Nerve Fiber after SCI

**Figure 6:**
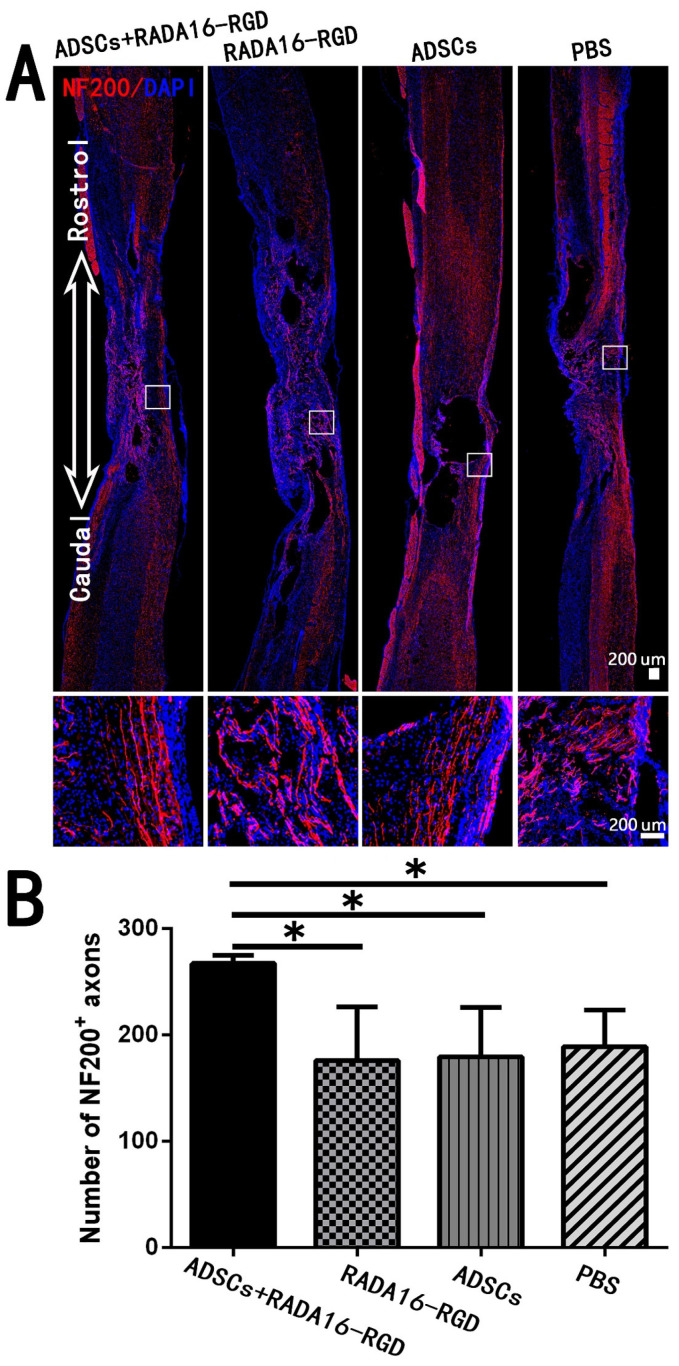
ADSCs+RADA16-RGD transplantation increases the number of NF200^+^ nerve fibers at the injury center. (**A**) At 8 weeks of transplantation of ADSCs+RADA16-RGD, NF200 antibody staining (red) was performed via passing through the central sagittal section of the spinal cord injury. The panels below (**A**) show high magnification of boxed regions in the top panels. (**B**) Quantitative analysis of NF200^+^ nerve fibers at the injury center. The ADSCs+RADA16-RGD group was significantly higher than the PBS, ADSCs, and RADA16-RGD groups (ADSCs+RADA16-RGD vs. PBS, ADSCs, or RADA16-RGD: *p* < 0.05). * *p* < 0.05. Data are presented as mean ± SEM (*n* = 3 per group). Scale bar = 200 µm. ADSCs: adipose stem cells; NF200: neurofilament 200; DAPI: 4′6-diamidino-2-phenylindole. NF200 (red): Alexa Fluor 647.

The most fundamental cause of motor and sensory dysfunction below the injury level after SCI is nerve fiber damage at the injury site, which interrupted the conduction pathway. The key to SCI treatment was to repair the interrupted nerve fibers and restore their conduction function. In the present study, we counted the NF200^+^ and 5-HT^+^ nerve fibers that were closely related to motor functions. The number of NF200^+^ fibers in the SCI center was significantly higher (Figure 6).

**Figure 7:**
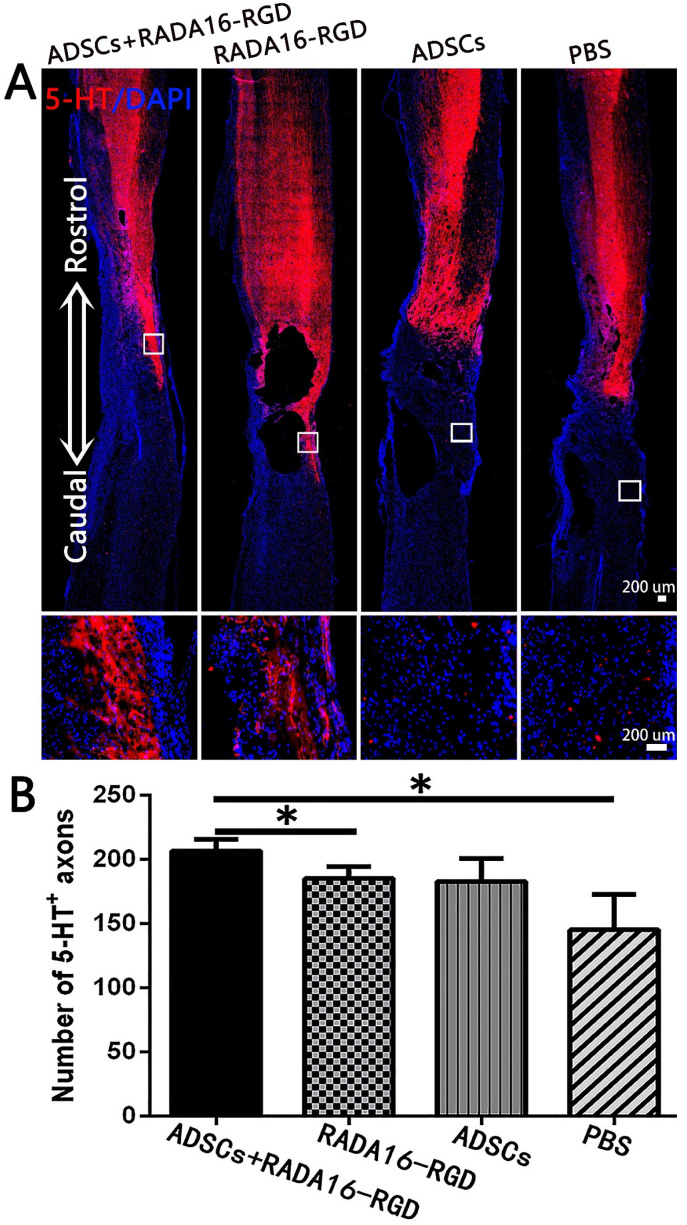
ADSCs+RADA16-RGD transplantation increases the number of 5-HT+ nerve fibers. (**A**) At 8 weeks of transplantation of ADSCs+RADA16-RGD, 5-HT antibody staining (red) was performed via passing through the central sagittal section of the spinal cord injury. The panels below (**A**) show high magnification of boxed regions in top panels. (**B**) Quantitative analysis of 5-HT^+^ nerve fibers at the injury center. The ADSCs+RADA16-RGD group was significantly higher than in the PBS and RADA16-RGD groups (ADSCs+RADA16-RGD vs. PBS or RADA16-RGD: *p* < 0.05). No 5-HT^+^ nerve fibers could be found in the three groups below the caudal edge of the injury center. * *p* < 0.05. Data are presented as mean ± SEM (*n* = 3 per group). Scale bar = 200 µm. ADSCs: adipose stem cells; 5-HT:5-hydroxy tryptamine; DAPI: 4′6-diamidino-2-phenylindole. 5-HT (red): Alexa Fluor 647.

The number of 5-HT^+^ fibers in the injury center was significantly higher in the ADSCs+RADA16-RGD (Figure 7).

### 3.8. Transplantation of ADSCs+RADA16-RGD Inhibited the Macrophage/Microglia Activation

**Figure 8:**
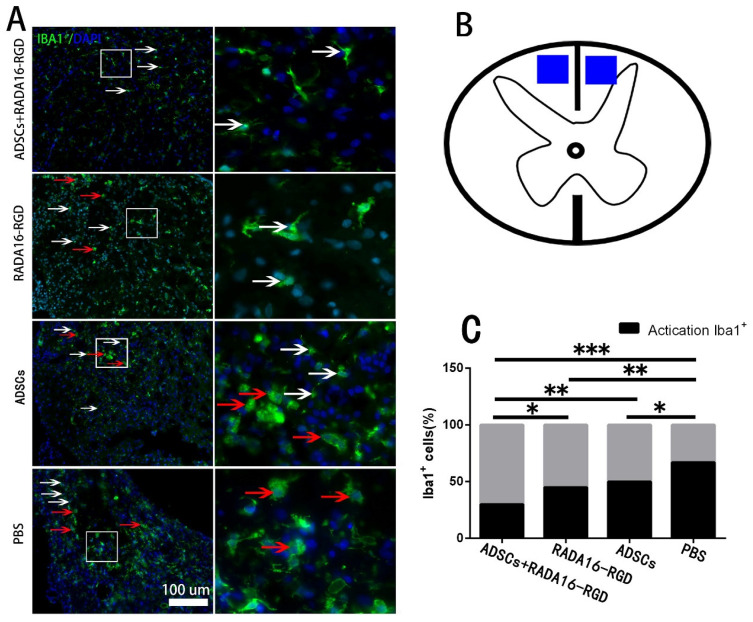
ADSCs+RADA16-RGD inhibited macrophage/microglial activation. (**A**) At 8 weeks of ADSCs+RADA16-RGD transplantation, Iba1 antibody (green) staining at 4 mm cephalic to the injury center was performed. Inactivated Iba1^+^ cells were smaller and shaped like stars with elongated branches (white arrows), while the activated cells were larger and in a round shape with fewer branches (red arrows). The right image is a high magnification of the corresponding area of the left image. (**B**) The blue area is the area where the cells are counted. (**C**) Statistics of Iba1^+^ cell activation rate at 4 mm cephalic to the injury center. The activation rate of Iba1^+^ cells (activated Iba1^+^ cells/Iba1^+^ cells) at 4 mm on the cephalic side of the injury center in the ADSCs+RADA16-RGD group was significantly lower than that of the PBS, ADSCs, and RADA16-RGD groups (ADSCs+RADA16-RGD vs. PBS: *p* < 0.001, ADSCs+RADA16-RGD vs. ADSCs: *p* < 0.01, ADSCs+RADA16-RGD vs. RADA16-RGD: *p* < 0.05), while that of the ADSCs and RADA16-RGD groups was significantly lower than that of the PBS groups (RADA16-RGD vs. PBS: *p* < 0.01, ADSCs vs. PBS: *p* < 0.05). * *p* < 0.05, ** *p* < 0.01, *** *p* < 0.001. Data are presented as n% (*n* = 4 per group). Scale bar = 100 µm. ADSCs: adipose stem cells; Iba1: ionized calcium-binding adapter molecule 1; DAPI: 4′6-diamidino-2-phenylindole. Iba1 (green): Alexa Fluor 488.

Macrophage/microglia played a key role in the inflammation resulting from chronic SCI. To investigate the effect of ADSCs+RADA16-RGD on macrophage/microglial activation, macrophage/microglia on the cephalic side of the injured center (4 mm distance) were stained with Iba1 to measure their activation 8 weeks after the transplantation. The activated Iba1^+^ cells possessed a large cell body and fewer and shorter processes. On the other hand, the non-activated Iba1^+^ cells had smaller cell bodies and slender processes. The activation rate of Iba1^+^ cells (activated Iba1^+^ cells/Iba1^+^ cells) at 4 mm on the cephalic side of the injury center was significantly lower in the ADSCs+RADA16-RGD group at 8 weeks of the transplantation (Figure 8).

### 3.9. Transplantation of ADSCs+RADA16-RGD Inhibited the Expression of Inflammatory Cytokines

**Figure 9:**
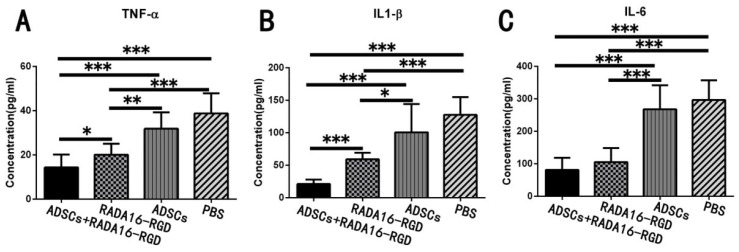
ADSCs+RADA16-RGD transplantation inhibits the expression of inflammatory factors. The spinal cord samples were collected from rats 2 weeks after ADSCs+RADA16-RGD transplantation to detect the expression of inflammatory cytokines TNF-α, IL-1β, and IL-6. (**A**) The expression of TNF-α was significantly lower in the ADSCs+RADA16-RGD group than in the PBS, ADSCs, and RADA16-RGD groups (ADSCs+RADA16-RGD vs. RADA16-RGD: *p* < 0.05, ADSCs+RADA16-RGD vs. ADSCs or PBS: *p* < 0.001). Moreover, the expression of TNF-α was significantly lower in the RADA16-RGD group than in the PBS and RADA16-RGD groups (RADA16-RGD vs. PBS: *p* < 0.001, RADA16-RGD vs. ADSCs: *p* < 0.01). (**B**) The expression of IL-1β was significantly lower in the ADSCs+RADA16-RGD group than in the PBS, ADSCs, and RADA16-RGD groups (ADSCs+RADA16-RGD vs. PBS, ADSCs, or RADA16-RGD: *p* < 0.001). Moreover, the expression of IL-1β was significantly lower in the RADA16-RGD group than in the PBS and ADSCs groups (RADA16-RGD vs. PBS: *p* < 0.001, RADA16-RGD vs. ADSCs: *p* < 0.05). (**C**) The IL-6 was significantly lower in the ADSCs+RADA16-RGD transplantation group than in the PBS and ADSCs groups (ADSCs+RADA16-RGD vs. PBS or ADSCs: *p* < 0.001), while it was significantly lower in the RADA16-RGD group than in the PBS and ADSCs groups (RADA16-RGD vs. PBS or ADSCs: *p* < 0.001). * *p* < 0.05, ** *p* < 0.01, *** *p* < 0.001. Data are presented as mean ± SEM (*n* = 3 per group). ADSCs: adipose stem cells; TNF-α: tumor necrosis factor α; IL-6: interleukin-6; IL-1β: interleukin-1 beta.

Acute inflammatory reaction after SCI results in neuronal death and axonal degeneration and atrophy, while chronic inflammation leads to neurodegeneration, glial scarring, and cavity formation. TNF-α, IL-1β, and IL-6 are highly expressed in the early stages of SCI and represent the inflammatory immune microenvironment. The expression levels of inflammatory cytokines, including TNF-α, IL-1β, and IL-6, were measured for 2 weeks and were significantly lower after the transplantation of ADSCs+RADA16-RGD (Figure 9).

### 3.10. LC-MS Analysis

**Figure 10:**
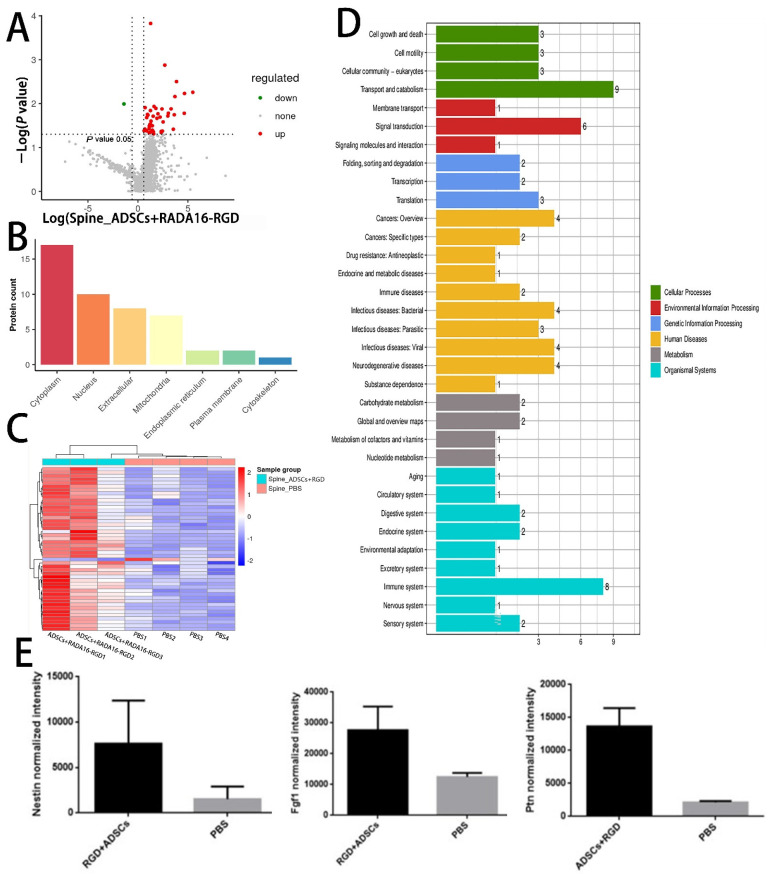
Differentially expressed proteins in the spinal cord of rats 2 weeks after ADSCs+RADA16-RGD transplantation. (**A**) Volcano plot of the proteins detected by liquid chromatography–mass spectrometry (LC-MS). The red dots represent the upregulated proteins and the green dots represent the downregulated proteins. (**B**) The subcellular localization of the differentially expressed proteins. (**C**) Heat map showing the distribution of differentially expressed proteins. Red indicates high expression and blue indicates low expression levels. (**D**) Gene Ontology term enrichment of differentially expressed proteins. (**E**) Relative expression of nestin, FGF1, and PTN in LC-MS analysis of the ADSCs+RADA16-RGD group and PBS group.

To further explore the potential molecular mechanism of ADSCs+RADA16-RGD action in SCI repair, we performed LC-MS analysis on the spinal cord tissues around the injured site in both the ADSCs+RADA16-RGD and PBS groups after 2 weeks of transplantation. A total of 3045 proteins were identified, of which 47 (Supplementary Table S2) were found to be differentially expressed. Of the differentially expressed proteins, 46 were upregulated and one was downregulated. These differentially expressed proteins are illustrated in the volcano plot (Figure 10A).

**Figure 11:**
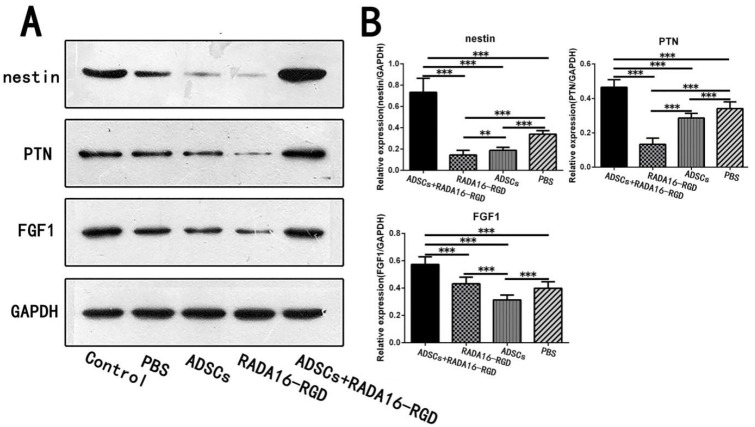
Expression of the selected proteins in week 2 as assessed by Western blotting. (**A**) Expression of nestin, FGF1, and PTN proteins in the second week by Western blotting. (**B**) Quantification of the relative protein expression. The expression of nestin in the ADSCs+RADA16-RGD group was significantly higher than that in the PBS, ADSCs, and RADA16-RGD groups (ADSCs+RADA16-RGD vs. PBS or ADSCs or RADA16-RGD: *p* < 0.001), while it was significantly lower in the RADA16-RGD group than in the ADSCs and PBS groups (RADA16-RGD vs. ADSCs: *p* < 0.01, RADA16-RGD vs. PBS: *p* < 0.001), and significantly lower in the ADSCs group compared to the PBS group (ADSCs vs. PBS: *p* < 0.001). The expression of PTN was significantly higher in the ADSCs+RADA16-RGD group than in the PBS, ADSCs, and RADA16-RGD groups (ADSCs+RADA16-RGD vs. PBS or ADSCs or RADA16-RGD: *p* < 0.001), significantly lower in the RADA16-RGD group than in the ADSCs and PBS groups (RADA16-RGD vs. ADSCs or PBS: *p* < 0.001), and significantly lower in the ADSCs group than in in the PBS group (ADSCs vs. PBS: *p* < 0.001). The expression of FGF1 was significantly higher in the ADSCs+RADA16-RGD group than that in the PBS, ADSCs, and RADA16-RGD groups (ADSCs+RADA16-RGD vs. PBS or ADSCs or RADA16-RGD: *p* < 0.001), and significantly lower in the ADSCs group than that in the RADA16-RGD and PBS groups (ADSCs vs. PBS or RADA16-RGD: *p* < 0.001). ** *p* < 0.01, *** *p* < 0.001. Data are presented as mean ± SEM (*n* = 3 per group). ADSCs: adipose stem cells; FGF1: fibroblast growth factor 1; PTN: pleiotrophin; GAPDH: glyceraldehyde-3phosphate dehydrogenase.

The heat map generated from the KEGG pathway analysis is presented in Figure 10C. Among the differentially expressed proteins, the expressions of pleiotrophin (PTN), fibroblast growth factor-1 (FGF1), and nestin, all of which are related to axonal growth and nervous system development, were upregulated in the ADSCs+RADA16-RGD group (Figure 10E). To verify the results of the LC-MS analysis, the expressions of PTN, FGF1, and nestin were determined by Western blotting. The expressions of nestin, PTN, and FGF1 were significantly higher in ADSCs+RADA16-RGD transplantation after 2 weeks (Figure 11).

## 4. Discussion

Axon conduction is interrupted after SCI. Therefore, the reconstruction of the interrupted conduction pathway and the restoration of the conduction function above and below the damaged plane are the key strategies for SCI repair. With the development of newer, high-performing biomaterials, it is now possible for new biomaterials to induce tissue and cell regeneration as well as to promote the repair of SCI. The application of nano-hydrogels in cell therapy and tissue engineering has provided broad prospects for neural regeneration. Rada16-rgd rapidly changes from the liquid form to gel after pH adjustment and binds directly to the host tissues. Peptide nanofiber hydrogels possess mechanical properties matching those of the extracellular matrix of the central nerve, and they can hence promote the growth of axons. This material also provides the best three-dimensional microenvironment for cells [31]. RADA16-RGD demonstrated good biocompatibility and exhibited effective controlled release of vascular endothelial growth factor (VEGF) and bone morphogenetic protein 2 (BMP-2). More importantly, when compared with RADA16-RGD loaded with a single growth factor or without growth factors, RADA16-RGD loaded with two growth factors exhibited a stronger ability to promote cell proliferation and osteogenesis [32]. The combination of RADA16-RGD and RADA16-IKAVA alleviated acute brain injury and promoted functional recovery in mice with intracerebral hemorrhage by reducing apoptosis, glial reaction, and inflammatory responses [33,34]. In peripheral nerve treatment, combined transplantation with RADA16-RGD and RADA16-IKAVA stimulated the regenerated nerve fibers to grow into the hydrogel and the Schwann cells to migrate into the hydrogel, thereby enhancing the recovery of motor functions [35]. ASCs derived from the adipose tissues are easily obtained when compared to other stem cells and promote tissue regeneration and repair by secreting cytokines and growth factors [36]. Cytokines and growth factors secreted by ASCs are involved in immune regulation (hepatocyte growth factor, HGF; prostaglandin E2, PGE2; transforming growth factor-β, TGF-β and IL-6), angiogenesis (FGF-2, HGF, VEGF, TGF-β2, and bFGF), nerve regeneration (glial cell line-derived neurotrophic factor, GDNF; nerve growth factor, NGF; insulin-like growth factor-1, IGF-1; brain-derived neurotrophic factor, BDNF), hematopoietic (HGF; granulocyte macrophage colony-stimulating factor, Gm-csf; IL-6I and IL-11), and other factors (adiponectin; angiotensin and chemokine (C-X-C motif) ligand 12, CXCL12) [37,38,39,40,41]. ADSCs can be used for the transplantation treatment of various tissues and organ injuries. For example, an intramuscular injection of ASCs into the plantar muscle can improve the burn-induced apoptosis of spinal cord ventral horn motor neurons and sciatic nerve Schwann cells as well as inhibit gastrocnemius atrophy [42]. Pre-differentiated human ASCs can be transformed into motor neuron-like cells with electrophysiological functions in vitro, which can be functionally integrated with the spinal cord tissues of the host after transplantation and establish synaptic connections with endogenous neurons to directly participate in the reconstruction of neural circuits at the damaged site [43]. Ngn2 gene knockout ADSCs implanted into the injured spinal cord can differentiate into NeuN^+^ and TUJ-1^+^ neurons, inhibit glial scar formation, and upregulate the VEGF and BNDF expression [44].

Our previous physical analysis of RADA16-RGD exposed that the self-assembled RADA16-RGD hydrogel possessed elasticity similar to that of the spinal cord tissue and could be transplanted to the injury site by injection [31]. The RADA16-RGD complex exhibited good biocompatibility and adhesion to the cells without facing immune rejection and promoted the proliferation and growth of neural stem cells [17]. In our previous study, the 3D culture of neural stem cells with RADA16-RGD promoted both cell proliferation and differentiation, indicating that the gel structure formed by RADA16-RGD allowed oxygen and nutrient transmission in the 3D environment [17]. When compared with the sole injection of ADSCs into the injured area, the number of viable ADSCs in the surrounding spinal cord tissue was higher after the transplantation of ADSC-RADA16-RGD into the cavity. This result confirms that the RADA16-RGD hydrogel could improve the survival of the ADSC stem cells in harsh environments [45]. We thus confirmed no significant difference between ADSCs and ADSCs+RADA16-RGD groups in vitro, although the ADSCs+RADA16-RGD group showed significantly higher functional recovery in vivo than the ADSCs group. This difference can be attributed to the difference in the survival number of ADSCs between the two groups in an in vivo microenvironment. In vitro, the two groups shared the same microenvironment, and there was no difference in the number of ADSCs. The RADA16-RGD sequence binds specifically to the integrins in the cell membrane, which promotes cell adhesion. Cells previously transplanted with ADSC-RADA16-RGD 1–4 weeks earlier were observed in the cavity, suggesting that RADA16-RGD endowed RADA16-I with the ability to promote cell adhesion without changing its biological properties. Furthermore, ADSCs+RADA16-RGD could inhibit the inflammatory reaction in the early stages of SCI, and 2 weeks after transplantation, the numbers of inflammatory cytokines TNF-α, IL-1β, and IL-6 were significantly lower. After ADSCs+RADA16-RGD transplantation eight weeks, the number of NeuN^+^ and ChAT^+^ neurons increased around the injury site. Moreover, few NeuN^+^ or ChAT^+^ neurons survived within 3 mm of the injury center, which may be because the intervention was performed 14 days after the injury. Both mechanical injury and acute inflammatory reaction in this stage could have led to neuronal death at the injury site, which needs to be confirmed in future studies. Myelination plays an important role in axonal conduction and motor function improvement. Past studies have demonstrated that the conduction velocity of axons increases by more than threefold when the demyelinated axons are remyelinated after glial cell transplantation [46]. ADSCs+RADA16-RGD can increase the MBP^+^ area around the injury and stimulate axon remyelination. At the injury center, although the MBP^+^ area was larger than that in the RADA16-RGD, ADSCs, and PBS groups, the differences were not statistically significant. We thus speculated that the degree of myelination is related to time. In future studies, we intend to extend the experiment to exclude the effect of time on axon myelination.

Reestablishing the interrupted neural pathway is pertinent for the successful repair of SCI. Our results for NF200^+^ nerve fibers suggest that ADSCs+RADA16-RGD could increase the numbers of these fibers at the injury center in relation to the inhibition of inflammation by ADSCs+RADA16-RGD, thereby alleviating the damage to the microenvironment. It has been reported that ADSCs can secrete various factors such as HGF and PGE2 as a part of regulating inflammation [47,48]. However, after 2 weeks of ADSC transplantation, the inflammatory cytokines TNF-α, IL-1β, and IL-6 were lower in expression, and no significant difference was noted with the PBS group. Accordingly, we hypothesized that the low survival rate of ADSCs after transplantation occurs because of the bad microenvironment at the injured site. We also found that after transplantation with RADA16-RGD, the levels of inflammatory cytokines were significantly lower than in the PBS and ADSCs groups, suggesting that RADA16-RGD could regulate the inflammatory microenvironment. However, the specific mechanisms behind this phenomenon warrant further study.

The 5-HT^+^ fibers in the spinal cord originate from the raphe nucleus and are widely distributed in the spinal cord. Past studies have demonstrated that 5-HT controls motor coordination and rhythm through a central pattern generator [49]. After SCI, the destruction of 5-HT^+^ fibers leads to 5-HT depletion, which induces different degrees of motor dysfunction and even paralysis [50]. In our study, no 5-HT^+^ nerve fibers were detected in the area below the caudal side of the injury center (Figure 8). Therefore, during the function scoring, although ADSCs+RADA16-RGD improved the motor function of the rats, the coordination was not significantly restored. These results suggest that, in addition to promoting the regeneration of NF200^+^ nerve fibers, the effects of 5-HT^+^ nerve fiber regeneration on functional recovery should be considered in the repair of severe SCI.

LC-MS analysis disclosed that ADSCs+RADA16-RGD promotes the expression of FGF1 and PTN, which are involved in neural plasticity and axon growth. PTN belongs to the heparin-binding metaphase factor family and is a secretory growth factor involved in the regulation of development. PTN plays several roles in neurodevelopment, including cell proliferation, neuronal migration, and neurite growth [51]. Moreover, PTN can transform the inhibitory effect of chondroitin sulfate proteoglycan on neurite growth into activation, which can significantly enhance nerve regeneration in mice with spinal cord transection [52]. FGF1 participates in the regulation of various developmental activities, including morphogenesis, cell differentiation, proliferation, and migration, and plays a crucial role in neuronal protection and axonal regeneration [53]. Moreover, FGF1 can increase the number of regenerated axons in the damaged vagus nerve and the number of motor neurons in the dorsal nucleus of the vagus nerve [54]. In addition, FGF1 can enhance the regeneration and survival of somatic motor and sensory neurons. For instance, FGF1 enhances axonal regeneration in the injured sciatic nerve [55].

## Data Availability

The data presented in this study are available in Supplementary Material here.

## Supplementary Materials

The following supporting information can be downloaded at: https://www.mdpi.com/article/10.3390/biology11050781/s1, Figure S1: ADSCs identification, Table S1: Sample size of rats in this experiment, Table S2: Differentially expressed proteins.
