# Supplementary material for: Human Adipose-Derived Stem Cells Combined with Nano-Hydrogel Promote Functional Recovery after Spinal Cord Injury in Rats
Source: Biology (Basel). 2022 May 20;11(5):781. doi: 10.3390/biology11050781 (PMC9138297; doi:10.3390/biology11050781)
Supplement: Supplementary file 1 [file biology-11-00781-s001.zip › biology-1699759-supplementary.pdf]

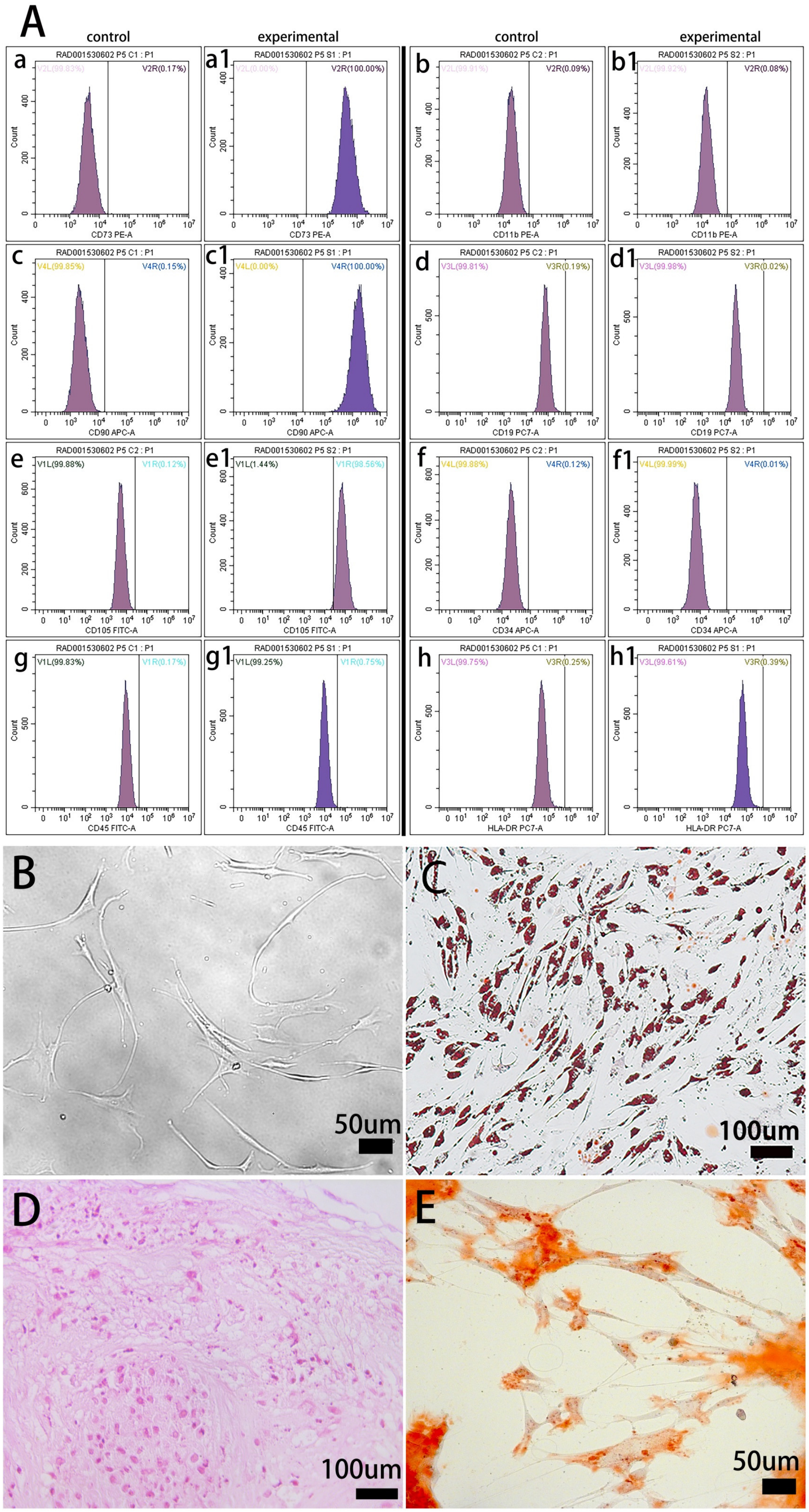
A
control
experimental
control
experimental
a
a1
b
b1
c
c1
d
d1
e
e1
f
f1
g
g1
h
h1
RAD001530602 P5 C1 : P1
RAD001530602 P5 S1 : P1
RAD001530602 P5 C2 : P1
RAD001530602 P5 S2 : P1
V2L(99.83%)
V2R(0.17%)
V2L(0.00%)
V2R(100.00%)
V2L(99.91%)
V2R(0.09%)
V2L(99.92%)
V2R(0.08%)
V4L(99.85%)
V4R(0.15%)
V4L(0.00%)
V4R(100.00%)
V3L(99.81%)
V3R(0.19%)
V3L(99.98%)
V3R(0.02%)
V1L(99.88%)
V1R(0.12%)
V1L(1.44%)
V1R(98.56%)
V4L(99.88%)
V4R(0.12%)
V4L(99.99%)
V4R(0.01%)
V1L(99.83%)
V1R(0.17%)
V1L(99.25%)
V1R(0.75%)
V3L(99.75%)
V3R(0.25%)
V3L(99.61%)
V3R(0.39%)
Count
CD73 PE-A
CD11b PE-A
CD90 APC-A
CD19 PC7-A
CD105 FITC-A
CD34 APC-A
CD45 FITC-A
HLA-DR PC7-A
B
50um
C
100um
D
100um
E
50um

Figure S1. ADSCs identification. A: The fifth-generation ADSCs flow cytometry analysis showed that the expression rates of mesenchymal stem cell positive markers CD73 (a, a1), CD90 (c, c1), and CD105 (e, e1) exceeded 95%, and the expression rates of cell negative markers CD11b (b, b1), CD19 (d, d1), CD34 (f, f1), CD45 (g, g1), and HLA-DR (h, h1) were less than 2%, indicating high cell purity. **B:** The shape of the fifth-generation of ADSCs was observed. They had long fusiform cell bodies with elongated processes on the surface. **C:** Adipose stem cells induce differentiation into adipocytes (Oil Red O).**D**: Adipose stem cells induce differentiation into cartilage(Safranin O). **E:** Osteogenic differentiation of adipose stem cells (Alizarin Red).B-E showed that transplanted cells could differentiate into adipocytes, chondrocytes, and osteocytes, which were consistent with the differentiation potential of ADSCs. B,E scale bars=50 um, C,D scale bars=100 um. ADSCs: Adipose stem cells.

Table S1. Sample size of rats in this experiment.

**Groups**	**AD-SCs+RADA16-RGD**	**ADSCs**	**RADA16-RGD**	**PBS**
Survival of transplanted cells	*n* = 12 (1W: *n* = 3, 2W: *n* = 3, 3W: *n* = 3, 4W: *n* = 3)	*n* = 12 (1W: *n* = 3, 2W: *n* = 3, 3W: *n* = 3, 4W: *n* = 3)		
IHC: *n* = 12 (BBB score:*n* = 8, and MEP:*n* = 4)	*n* = 12 (Chat,NeuN,MBP:*n* = 6, NF-200:*n* = 3, 5-HT: *n* = 3)	*n* = 12 (Chat,NeuN,MBP: *n* = 6, NF-200: *n*=3, 5-HT: *n*=3)	*n* = 12 (Chat,NeuN,MBP: *n* = 6, NF-200: *n*=3, 5-HT: *n* = 3)	*n* = 12 (Chat,NeuN,MBP: *n* = 6, NF-200: *n* = 3, 5-HT: *n* = 3)
Iba1(Additional experiments)	*n* = 4	*n* = 4	*n* = 4	*n* = 4
Elisa	*n* = 3	*n* = 3	*n* = 3	*n* = 3
LC-MS	*n* = 3			*n* = 4
WB	*n* = 3	*n* = 3	*n* = 3	*n* = 3
Totle	*n* = 37	*n* = 34	*n* = 22	*n* = 26

Table S2. Differentially expressed proteins.

Protein	Spine_CR	Spine_CR	Spine_CR	Spine_PB	Spine_PB	Spine_PB	Spine_PB	ratio	t.test_p	regulated	ProteinG	Descript	Gene Name
A1L1K3	135991.4	92439.41	140162	49783.64	59091.57	70259.97	74145.32	1.940367	0.017623	up	A1L1K3	Anaphase	Anapc5
A6JFQ6	29468.32	7001.709	15066.58	482.1649	495.3949	3675.246	627.2869	13.01407	0.006915	up	A6JFQ6	Clavesin	Clvs1
A8IHN8	44628.94	22507.18	30720.49	13123.52	6330.119	4349.643	13764.37	3.47308	0.012828	up	A8IHN8	Uncharac	Ag2
D3ZCL3	16765.17	4557.034	16571.18	482.1649	495.3949	427.0003	627.2869	24.8663	0.016623	up	D3ZCL3	U1 small	Snrpc
F2Z3T4	19146.59	8733.67	12788.2	5463.348	5278.116	4329.99	7540.828	2.398016	0.044405	up	F2Z3T4	Musclebl	Mbnl2
I6L9G5	5546.073	3875.812	2604.873	482.1649	2008.06	1496.827	1976.696	2.688859	0.045747	up	I6L9G5	Reticulo	Rcn3
O35162	58280.23	24032.27	34479.1	12745.63	17165.98	9994.994	18279.85	2.676261	0.044492	up	O35162	Heat sho	Hspa13
P00697	71629.73	37061.13	95105.11	13355.8	21239.38	10034.61	48517.13	2.917198	0.043873	up	P00697	Lysozyme	Lyz1
P07150	106962.7	65873.28	90720.25	54862.24	46836.92	35409.22	38592.25	2.00004	0.018253	up	P07150	Annexin	Anxa1
P0C0A1	40537.67	21251.03	40560.63	9998.23	16480.07	17393.15	18614.88	2.18393	0.046104	up	P0C0A1	Vacuolar	Vps25
P10687	27622.67	13924.62	19959.01	6377.44	8421.779	8714.209	10994.48	2.376511	0.02933	up	P10687	1-phosph	Plcb1
P21263	12259.22	2772.578	7864.301	1781.64	495.3949	427.0003	3373.484	5.023123	0.04295	up	P21263	Nestin O	Nes
P25886	28636.76	12475.24	24918.75	6209.79	8254.675	5059.509	14986.79	2.551117	0.043206	up	P25886	60S ribo	Rpl29
P29067	16784.9	11221.06	18234.55	482.1649	6205.827	427.0003	627.2869	7.963288	0.019137	up	P29067	Beta-arr	Arrb2
P31721	82565.71	38834.77	49534.78	21538.38	21315.88	10301.43	25950.42	2.881114	0.019339	up	P31721	Compleme	C1qb
P43527	11962.46	5030.025	7498.847	700.7345	495.3949	427.0003	627.2869	14.5107	0.003146	up	P43527	Caspase-	Casp1
P52925	28622.38	20317.72	24093.61	11602.87	17624.33	10880.49	19832.61	1.624588	0.037907	up	P52925	High mob	Hmgb2
P53676	76374.44	11329.86	11389.22	482.1649	6665.519	427.0003	3760.997	11.65565	0.038353	up	P53676	AP-3 com	Ap3m1
P53678	38958.14	22636.22	21236.26	16292.77	9774.99	11363.83	13536.36	2.166868	0.037073	up	P53678	AP-3 com	Ap3m2
P55009	40269.29	19040.01	49323.45	15760.83	9004.182	5205.61	8878.083	3.728403	0.021661	up	P55009	Allograf	Aif1
P61149	26987.73	20313.71	35639.79	11827.54	11521.43	12095.04	14361.88	2.220386	0.03189	up	P61149	Fibrobla	Fgf1
P62268	52373.19	26461.93	38825.78	10879.67	13385.1	20621.35	25508.82	2.228586	0.033668	up	P62268	40S ribo	Rps23
P62634	32957.89	15676.14	38794.39	7887.914	10608.63	7682.85	2861.571	4.014027	0.017035	up	P62634	Cellular	Cnbp
P63055	158650.7	77006.31	179957.7	76106.38	44549.22	32777.82	49760.24	2.727216	0.040377	up	P63055	Calmodul	Pcp4
P63090	15115.84	10451.53	15395.6	1982.443	2281.363	1864.246	2342.967	6.447547	0.001329	up	P63090	Pleiotro	Ptn

P63255	13210.83	24048.23	76798.18	482.1649	495.3949	4413.16	627.2869	25.27022	0.005869	up	P63255	Cysteine	Crip1
P84817	350387.1	187216.9	306172.9	122481.2	122881.5	91106.88	150344.6	2.311017	0.029535	up	P84817	Mitochon	Fis1
P97576	17860.01	16802.59	17176.43	6226.554	7533.024	7023.718	7895.626	2.410087	0.000149	up	P97576	GrpE pro	Grpel1
P97675	38504.7	17278.65	12728.37	482.1649	495.3949	427.0003	627.2869	44.95858	0.00552	up	P97675	Ectonucl	Enpp3
Q09167	51549.31	29803.89	62305.52	13842.25	495.3949	427.0003	627.2869	12.4445	0.017973	up	Q09167	Serine/a	Srsf5
Q498U4	36955.12	18615.96	28932.48	11628.22	10932.73	8076.49	6254.093	3.054127	0.011603	up	Q498U4	SAP doma	Sarnp
Q4KLH4	65837.23	33142.56	38940.57	13641.58	15177.26	14310.71	31143.33	2.475921	0.024985	up	Q4KLH4	Paraspec	Pspc1
Q4KLK9	14277.07	5438.278	14911.18	6497.844	495.3949	427.0003	627.2869	5.737007	0.026154	up	Q4KLK9	RNA poly	Ssu72
Q4V7F2	39945	23904.4	25115.59	13651.89	10743.89	7824.226	17601.71	2.380889	0.014366	up	Q4V7F2	Cysteine	Creld1
Q5BK62	772.6278	466.7632	653.366	1735.385	1012.502	1476.121	2432.458	0.379132	0.010172	down	Q5BK62	Protein	Mpv17
Q63663	52909.4	18491.44	35680.04	7402.287	7277.684	4805.169	11278.32	4.641043	0.020291	up	Q63663	Guanylat	Gbp2
Q63691	24168.86	4449.944	15578.4	9232.824	495.3949	427.0003	627.2869	5.465299	0.042194	up	Q63691	Monocyte	Cd14
Q6AYZ1	675104.6	551522.7	1282772	120451.7	173817.7	143303.3	194669	5.292068	0.013214	up	Q6AYZ1	Tubulin	Tu-ba1c
Q6MG08	13573.85	9886.047	11999.16	7506.565	5847.306	6758.415	8680.214	1.642051	0.012336	up	Q6MG08	ATP-bind	Abcf1
Q80T18	51111.64	14495.84	18959.55	6435.278	4238.378	2385.85	627.2869	8.238309	0.016552	up	Q80T18	Glia mat	Gmfg
Q811M5	21341.25	11071.95	26620.71	482.1649	6368.159	427.0003	627.2869	9.957717	0.013167	up	Q811M5	Compleme	C6
Q923V8	23120.96	9990.439	23089.3	6285.189	5367.668	5070.832	8464.774	2.974944	0.048746	up	Q923V8	Selenopr	Sele-nof
Q9ES54	27388.6	19595.65	18762.68	10138.8	14435.43	8243.766	14967.16	1.834515	0.020802	up	Q9ES54	Nuclear	Nploc 4
Q9QY44	15885.14	6390.143	22121.84	6505.077	495.3949	4092.126	627.2869	5.050915	0.045807	up	Q9QY44	ATP-bind	Abcd2
Q9QYU2	35484	20682.23	31074.28	16648.87	12839.67	13016.38	21286.11	1.823465	0.041716	up	Q9QYU2	Elongati	Tsfm
Q9WTT2	18580.62	20564.74	15929.4	14658.67	13551.06	7829.308	11790.21	1.535316	0.041433	up	Q9WTT2	Caseinol	Clpb
Q9Z1M9	8267.983	3646.136	6814.98	4188.469	2005.565	1533.165	1475.354	2.713609	0.03188	up	Q9Z1M9	Structur	Smc1a
